# Vole abundance and reindeer carcasses determine breeding activity of Arctic foxes in low Arctic Yamal, Russia

**DOI:** 10.1186/s12898-017-0142-z

**Published:** 2017-09-16

**Authors:** Dorothee Ehrich, Maite Cerezo, Anna Y. Rodnikova, Natalya A. Sokolova, Eva Fuglei, Victor G. Shtro, Aleksandr A. Sokolov

**Affiliations:** 1 Department of Arctic and Marine Biology, University of Tromsø-The Arctic University of Norway, 9037 Tromsø, Norway; 2 Faculty of Biology, Lomonosov Moscow State University, GSP-1, Leninskie Gory, Moscow, 119991 Russia; 3 Arctic Research Station of Institute of Plant and Animal Ecology, Ural Branch, Russian Academy of Sciences, 629400, Zelenaya Gorka Str., 21, Labytnangi, Russia; 4 Arctic Research Center of Yamal-Nenets Autonomous District, Salekhard, Russia; 5 Norwegian Polar Institute, Fram Centre, PostBox 6606, Langnes, 9296 Tromsø, Norway

**Keywords:** Food web, Numerical response, Reindeer carcasses, Small rodent community, Vole cycle, Diet, *Vulpes lagopus*

## Abstract

**Background:**

High latitude ecosystems are at present changing rapidly under the influence of climate warming, and specialized Arctic species at the southern margin of the Arctic may be particularly affected. The Arctic fox (*Vulpes lagopus*), a small mammalian predator endemic to northern tundra areas, is able to exploit different resources in the context of varying tundra ecosystems. Although generally widespread, it is critically endangered in subarctic Fennoscandia, where a fading out of the characteristic lemming cycles and competition with abundant red foxes have been identified as main threats. We studied an Arctic fox population at the Erkuta Tundra Monitoring site in low Arctic Yamal (Russia) during 10 years in order to determine which resources support the breeding activity in this population. In the study area, lemmings have been rare during the last 15 years and red foxes are nearly absent, creating an interesting contrast to the situation in Fennoscandia.

**Results:**

Arctic fox was breeding in nine of the 10 years of the study. The number of active dens was on average 2.6 (range 0–6) per 100 km^2^ and increased with small rodent abundance. It was also higher after winters with many reindeer carcasses, which occurred when mortality was unusually high due to icy pastures following rain-on-snow events. Average litter size was 5.2 (SD = 2.1). Scat dissection suggested that small rodents (mostly *Microtus* spp.) were the most important prey category. Prey remains observed at dens show that birds, notably waterfowl, were also an important resource in summer.

**Conclusions:**

The Arctic fox in southern Yamal, which is part of a species-rich low Arctic food web, seems at present able to cope with a state shift of the small rodent community from high amplitude cyclicity with lemming dominated peaks, to a vole community with low amplitude fluctuations. The estimated breeding parameters characterized the population as intermediate between the lemming fox and the coastal fox ecotype. Only continued ecosystem-based monitoring will reveal their fate in a changing tundra ecosystem.

**Electronic supplementary material:**

The online version of this article (doi:10.1186/s12898-017-0142-z) contains supplementary material, which is available to authorized users.

## Background

Arctic ecosystems are at present changing rapidly under the influence of climate warming [1]. At the southern margin of the Arctic, temperatures now often exceed those characteristic for the Arctic [2], rain falling in winter hardens the snow pack [3], tall shrubs and boreal species expand, whereas typical Arctic species are impacted negatively [4, 5]. The Arctic fox (*Vulpes lagopus*) is a widespread and common Arctic predator, which is endemic to the circumpolar tundra areas. It has been chosen as one of ten flagship species for the impact of climate change highlighted by the International Union for Conservation of Nature [6], and the species is a good candidate to become the object of coordinated circumpolar monitoring [7] as asked for by the Arctic Terrestrial Biodiversity Monitoring Plan [8]. Given the potential vulnerability of the species to changes in the prey base and the availability of competitors [5], it is important to understand what drives the dynamics of Arctic fox populations in different ecological contexts, in particular in the low Arctic.

Adapted to survive scarcity and extreme cold in little productive ecosystems [9], Arctic foxes are able to exploit many different resources including lemmings (*Lemmus* sp. and *Dicrostonyx* sp.), birds, as well as marine resources or ungulate carcasses [10]. Depending on the prevailing prey base, two main ecotypes have been described [11, 12]: lemming foxes and coastal foxes. Lemming or inland foxes feed preferably on lemmings, but switch to alternative prey such as birds in low lemming years [13]. They maximize reproductive effort in peak years with very large litters, but may skip breeding or breed poorly in years with low lemming abundance [14, 15]. This results in clear population fluctuations, which follow the lemming cycle [16]. A recent survey of Arctic fox monitoring initiatives revealed that strong multiannual fluctuations were prevalent in the majority of populations, and that most of these populations feed on lemmings [7]. This was also the case on Yamal Peninsula, Russia, during the 1970s and 1980s, when large-scale surveys showed that Arctic fox den occupancy and the percentage of pregnant females among hunted foxes were related to lemming abundance [17].

Coastal foxes, on the contrary, rely mostly on more stable marine resources such as sea bird colonies. In coastal populations, for instance on Iceland or on Mednyi Island in the Bering Sea, breeding occurs nearly every year, but litters are smaller [11, 18]. In the high Arctic Svalbard archipelago, Norway, where native populations of small rodents are absent, Arctic foxes exploit sea birds, reindeer (*Rangifer tarandus*) carcasses and geese, with highest reproductive output in dens close to seabird colonies [19]. Year to year variation in den occupancy is mainly driven by the availability of reindeer carcasses in late winter [20, 21]. Substantial variation in the density of breeding pairs in the absence of small rodents was also observed on Kolguev Island, Russia, where Arctic foxes feed mainly on geese in summer [22]. Considering the varied resources used by non-lemming foxes and the diverse dynamics observed in such populations, Eide et al. [19] suggested moving beyond the distinction between lemming and coastal foxes when studying resource dependency of Arctic foxes, and rather investigating which main resources drive the fox dynamics in a specific ecosystem context (see also [23]).

The status of Arctic fox populations close to the southern margin of the Arctic tundra biome varies between areas depending on specific ecological processes. The coastal fox populations in Iceland, where red foxes (*Vulpes vulpes*) are absent, have been growing until recently due to an increase in carrying capacity attributed to growing populations of marine birds and geese [24, 25]. Mainland populations, on the contrary, are particularly exposed to increased pressure from competitively superior red foxes expanding northwards. This is the case in Fennoscandia, where Arctic foxes are critically endangered [26]. On Varanger Peninsula, northeastern Norway, two main drivers have been identified for the decline of the Arctic fox: (1) an increase in the population of red foxes, which is subsidized by carrion of semi-domestic reindeer [27]; and (2) a scarcity of lemmings due to increasing irregularity of their conspicuous peak years [5]. A fading out of the characteristic lemming cycles leading to low density populations with detrimental consequences for specialized Arctic predators has indeed been observed in several regions of the Arctic and attributed to changing winter climate [4, 28, 29]. Voles (*Myodes rutilus* and *Microtus oeconomus*), on the contrary, are abundant on Varanger Peninsula and exhibit population cycles with a period of 4–5 years [29]. However, they seem not able to replace lemmings as a resource, as Arctic fox reproduction did not respond to a vole peak without lemmings in 2015 [5]. Presently, little is known about the drivers of Arctic fox populations at the southern margin of the vast Eurasian tundra in Russia.

Here we present data from a 10-year study of Arctic fox at the Erkuta Tundra Monitoring site in Yamal, Russia. The aim of the study was to identify the main resources driving the dynamics of this low Arctic population, experiencing at present little competition from red foxes [30]. To answer this question, we first determined factors explaining variation in breeding activity, and second assembled available data about the diet of foxes during the breeding season and assessed whether it varied according to resource availability. We hypothesized that breeding productivity in this inland population would be related to the small rodent dynamics, in particular lemming abundance, as described for Fennoscandia [5, 31] and for southern Yamal during the 1980s [17]. However, we also assessed the importance of other resources available in the study area in late winter, at the time when Arctic fox females initiate breeding [19], such as willow ptarmigan (*Lagopus lagopus*), mountain hare (*Lepus timidus*) and reindeer carcasses.

## Methods

### Study area and components of the vertebrate food web

**Fig. 1:**
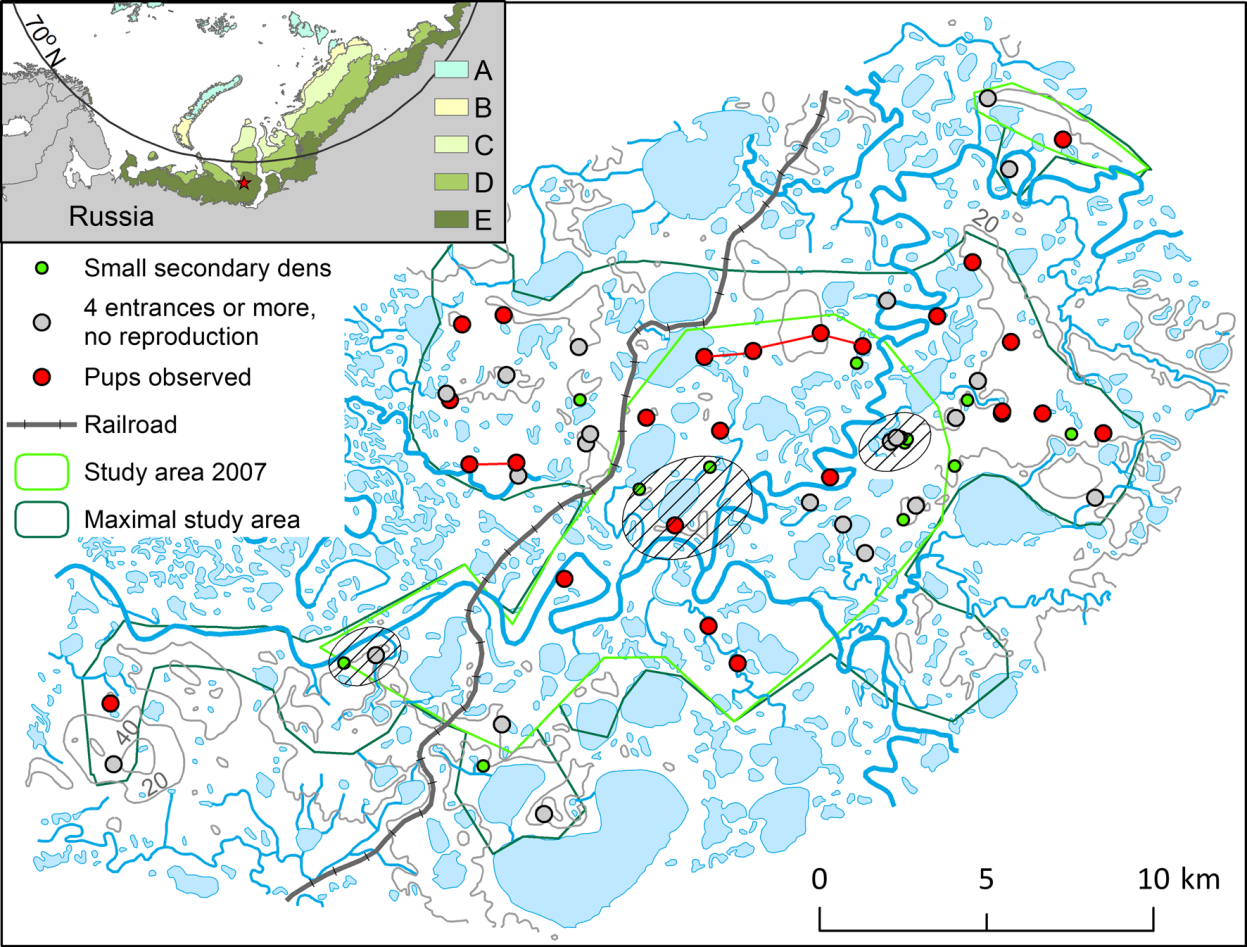
Map of the Erkuta tundra monitoring site in southern Yamal, Russia. The inset indicates the location of the area in the western Eurasian Arctic (represented by the red star) and shows the five bioclimatic subzones of the Arctic according to [2]. Subzones A–C represent the high Arctic whereas subzones D and E represent the low Arctic. The hatched ellipses show the three replicate areas (units), where herbivore faeces counts and small rodent trapping were carried out. All fox dens are shown. Red dots represent dens where pups have been observed during the study period, and red lines link dens between which fox families have moved. The green lines show the extent of the study area in the first year of the study (2007) and the maximal extent of the study area. Note, however, that in most years the area actually surveyed was somewhat less than this maximal area

The Erkuta Tundra Monitoring site is situated in the southern part of Yamal Peninsula (Russia) close to the confluence of the Payutayakha and Erkutayakha rivers (68.2°N, 69.2°E; Fig. 1). This low Arctic area is characterized by a tundra landscape with gently rolling hills (ca 30 m high), including some steep slopes and sandy cliffs along riverbanks and lakes. Mean temperature in the area is −24.1 °C in January and 11.4 °C in July, and mean annual precipitation is about 335 mm (averages for the period 1960–1990; from [32]). The substrate consists of sandy and clayey sediments that provide good opportunities for den excavation by Arctic foxes. Permafrost is continuous [33]. Numerous water bodies sustain extensive wetlands, and dense thickets of tall shrubs more than 2 m high (willows *Salix sp*. and some alder *Alnus fruticosa*) occur along rivers and lakes. The main vegetation consists of low shrub tundra and erect dwarf shrub tundra [34].

Arctic fox is the most common mammalian predator, but least weasel (*Mustela nivalis*), stoat (*M. erminea*), wolverine (*Gulo gulo*) and wolf (*Canis lupus*) are also present. Red foxes were rare in the beginning of the study in 2007 [35], but the first two breeding events were recorded in 2014 [30]. The most common birds of prey in the area are rough-legged buzzards (*Buteo lagopus*), peregrine falcons (*Falco peregrinus*), and long-tailed and Arctic skuas (*Stercorarius longicaudus* and *S. parasiticus*). Raven (*Corvus corax*) have been breeding in the study area since 2009 and we recorded hooded crow (*Corvus cornix*) breeding for the first time in 2014 [30]. In total, 40 species of migratory birds, including numerous passerines, waders and waterfowl, are breeding in the study area [36]. Several species of geese are present in rather low numbers, but ducks are numerous both as breeders and as non-breeders on rivers and lakes.

The vertebrate food web comprises many species of herbivores. The small rodent community consists of five species: Narrow-sculled voles (*Microtus gregalis*) and Middendorff’s voles (*M. middendorffii*) are most abundant. Other species are collared lemming (*Dicrostonyx torquatus*), Siberian lemming (*Lemmus sibiricus*), and red-backed vole (*Myodes rutilus*) [37]. During the last decade, small rodent fluctuations were of low amplitude and rather low densities [37]. The last high amplitude small rodent peak was recorded in 1999 [38], but earlier high amplitude population cycles with a period of 3–5 years were occurring in the area and lemmings were more common (Shtro [17]). The most abundant resident medium-sized herbivores are willow ptarmigan and mountain hare. The muskrat (*Ondatra zibethica*) is also present. Domestic reindeer, herded traditionally by Nenets people, are the only large herbivore. According to official statistics there were approximately 300,000 reindeer on Yamal Peninsula in 2013, and our study area is used by herds in all seasons. During the last decade, extensive ground icing in winter resulting from heavy rain-on-snow events (ROS [3]) caused unusually high reindeer mortality during two winters: 2006–2007 [39] and 2013–2014 [30]. The most recent event caused the death of 40,000 reindeer in Yamalskyi district according to official statistics and had dramatic consequences for local herders. The high availability of reindeer carrion in that year was confirmed by numerous observations of reindeer carcasses in our study area.

### Monitoring of herbivore populations

Small rodent dynamics were monitored from 2007 to 2016 by snap trapping, which was carried out according to the small quadrat method [40]. Three traps baited with raisins and rolled oats were placed at each corner of a 15 × 15 m quadrat for two nights in the second part of June and in the beginning of August (2 × 12 = 24 trap nights per quadrat). We placed quadrats in three habitats (willow thicket edge, wet tundra and dry tundra; see [37] for a description of the habitats) and replicated the design in two spatial units from 2007 to 2011, resulting in 2 × 3 × 6 = 36 quadrats in total (864 trap nights) per session. Since 2012 we surveyed three spatial units (54 quadrats = 1296 trap nights per session), and in 2007 and 2016 we carried out only one trapping session for logistic reasons. The data were summarized as total number of individuals of lemmings and voles respectively, trapped per 24 trap nights and averaged over units and habitats. We assumed that these indices reflected the relative changes in abundance over time of the respective small rodents in our study area, although they are not estimates of density.

We carried out faeces counts on permanent removal plots according to the same design as above to obtain an estimate of the relative presence and activity of ptarmigan and hare. The 15 × 15 m trapping quadrats were surrounded by eight small plots of 50 × 50 cm where faeces were counted [41]. The data were summarized as faeces occurrence, i.e. the number of small plots with presence of faeces among the eight small plots surrounding one quadrat, and averaged over habitats and units. This resulted in an index of overall relative presence of these species in the study area, similar to the small rodent index described above. In 2007, 2009 and 2016, because of logistic reasons, faeces were counted only once.

### Arctic fox den survey

From 2007 to 2016, we carried out systematic fox den surveys each summer. We started with a core area of ca 130 km^2^ in 2007, comprising most breeding dens that were known at that time [35]. In subsequent years, we progressively enlarged the study area and searched for more dens. By 2014, the area covered was 230 km^2^. To the extent possible given logistic constraints, we visited all known dens annually. Dens were first observed from a distance with binoculars to check for the presence of Arctic foxes. Subsequently, we walked to the den and recorded the number of entrances, whether these were cleaned and showed traces of recent digging, footprints, prey remains, and the presence of pups in the den (sounds). A den was considered active (i.e. inhabited by a breeding fox family) if pups were seen or clear sounds of pups were heard from the den. The minimum number of pups was determined for most breeding dens either by observing the den from a distance over a period of at least 5 h (or until an adult brought food and the pups emerged), or by using an automatic camera on the den. We fixed an automatic camera (Reconyx PC85/PC800; Reconyx Inc., Holmen, WI, USA) on wooden poles and placed it for a period of between 1 and 5 weeks at approximately 2–8 m from den entrances in a position providing a good overview of the den. The cameras used a motion sensor programmed to high sensitivity, and were taking ten pictures for each trigger. We visited active dens between one and five times during the summer.

### Arctic fox diet

We investigated the diet of Arctic foxes using three complementary methods: scat dissection, description of prey remains on active dens and analysis of stable isotopes. Scat dissection is likely to lead to an overrepresentation of small prey such as small rodents, which are consumed with identifiable bones and teeth, whereas larger prey such as hare or reindeer carcasses, from which mostly meat is consumed, are likely to be underrepresented. Larger prey, on the contrary, are better represented in the prey remains than small rodents, which are often consumed whole or taken into the den [22, 42]. Stable isotopes reflect the mixture of resources consumed over a certain period, and have a lower resolution than the two previous methods, because they can only distinguish between resources with distinct isotopic signatures [43].

#### Scat dissection

We collected fresh scats on breeding dens in the summers of 2007, 2013 and 2014, several times during the summer when possible. All scats were stored in a freezer at −80 °C before analysis to prevent human exposure to eggs from the tapeworm *Echinococcus multilocularis*. In 2007, scats from seven dens were obtained, and the total material collected on a den at a particular date was analyzed as one bulk sample. Scats were soaked in water, fragmented by hand and remains of rodents (fur, bones, teeth; including muskrat), birds (feathers and bones), reindeer (fur), hare (fur, identified by comparing with reference samples), fish, insects and plants were visually sorted, using a magnifying glass when necessary. Subsequently, remains were dried at 60 °C for 2 days, and weighed. Results were summarized as percent dry weight. In 2013 and 2014, a different protocol was used, because the data resulted from a different student project: 21 scats per den and collection date were analyzed individually [44]. After soaking in water with laundry soap, solid parts were separated by washing through a sieve (mesh size 0.5 mm). As above, the scat material was visually sorted into the categories small or medium sized mammal fur (small rodents, muskrat and hare), feathers, small rodent bones and teeth, bird bones, eggs, fish, insects and plants. We estimated percentage volume for the different remain categories according to the whole faeces equivalent approach of [15]. For all years, small rodent species were identified by examining the first molar of the lower jaw [45].

#### Prey remains

From 2010 to 2016, we recorded prey remains during visits to dens with clear signs of recent fox presence (but not only on breeding dens). On the first visit, we described all fresh remains, and removed them from the den. Items, which still contained food, were not removed, but registered in order to avoid counting them again on the next visit. Visits during which the observer did not pay attention to prey remains, but for instance only collected an automatic camera, were excluded from the analysis.

#### Stable isotopes

Winter fur was collected each summer at the entrance of dens or when encountered otherwise, and analyzed for stable isotopes of carbon (δ^13^C) and nitrogen (δ^15^N; see [10] for details about the methods). The fur shed in spring reflects the diet during the period when the foxes were molting to winter fur in the previous fall. Stable isotope signatures of the main prey species were available from the International Polar Year project “Arctic Predators” [10]. These consisted of muscle samples collected in summer or in fall primarily from 2007 to 2009 (see [10] for details about collecting tissue of the different species, and choosing and aggregating of prey signatures). A few additional prey signatures, notably from muskrat, were obtained more recently from samples collected opportunistically, for instance from remains on Arctic fox dens.

### Statistical analysis

Data analysis was carried out in R version 3.2.2 [46]. The probability of a den to be active in a particular year was analyzed with GLMMs with a logit link and a binomial error distribution using the function glmer of the package lme4 in R [47]. Den identity (hereafter “Den ID”) was included as a random effect in all models to account for differences in the quality of dens and the surrounding territories, and potentially important resources were considered as fixed effects. As lemmings are known to be a main driver of Arctic fox breeding dynamics (e.g. [14, 15], the average number of lemmings trapped in June was used as a proxy of lemming abundance in late winter when Arctic fox females initiate breeding. Because voles were much more abundant in the study area than lemmings, and the total amount of small rodents may be important, we also included the log of the average number of all small rodents trapped in June. Other resources present in late winter and considered possible determinants of breeding activity, were reindeer carcasses, ptarmigan and hare. Since no quantitative estimates of the number of reindeer carcasses were available for our study area, we used a factor with two levels: “high” abundance for the breeding seasons 2007 and 2014 (the 2 years where unusually high reindeer mortality was documented in the media and scientific literature [30, 39]) and “Usual” abundance of reindeer carcasses for the other years. For hare and ptarmigan, we used the average occurrence indices obtained from faeces counts in June, as the faeces, which accumulated since the previous August, reflect the presence of these herbivores in winter. We assembled a set of candidate models consisting of a model with an intercept only, and one or two additive fixed effects. We started with models including lemming or total small rodent abundance as the most likely driver of breeding activity, and then included other potential resources (reindeer, hare or ptarmigan). The most suitable model was chosen according to Akaike’s information criterion corrected for small sample sizes (AICc). Models with a difference in AICc (ΔAICc) of <2 were considered equally adequate. The selected model was graphically checked for constant variance of residuals, presence of outliers and normality of the random effects.

We used a similar modelling approach for the minimum number of pups at the dens. Here a GLMM with a log link and a Poisson error distribution was used. We included only data about the number of pups that had been estimated either by thorough observation or with automatic cameras. As above, lemmings, total small rodent abundance, reindeer, hare and ptarmigan were used as additive fixed effects in candidate models. Den ID was included as a random effect in all models.

We summarized the scat dissection data as mean proportions (either dry weight or volume) of different prey categories per year, and as frequencies of occurrence of the main prey types. The observations of prey remains were presented as the proportion of den visits carried out each year, at which a certain category of prey was observed. We carried out a correspondence analysis using the function dudi.coa from the R package ade4 [48] to assess differences between years in the occurrences of prey remains.

The stable isotope data of Arctic fox fur were presented graphically by plotting the fox values together with the mean signatures of main prey groups. The fox signatures were corrected for isotopic discrimination using the factors determined by [49]. Linear mixed effects models (LMM) were used to investigate whether the stable isotope signatures of winter fur varied with small rodent abundance in late summer. For each isotope, we implemented a model with the total trapping index of small rodents in August of the year preceding the fur sample collection as a fixed factor, and the collection place of the sample as a random factor. Analyses were carried out using the function lmer in lme4, and models were graphically checked for constant variance of residuals, presence of outliers and normality of the random effects.

## Results

### Herbivore dynamics

**Fig. 2:**
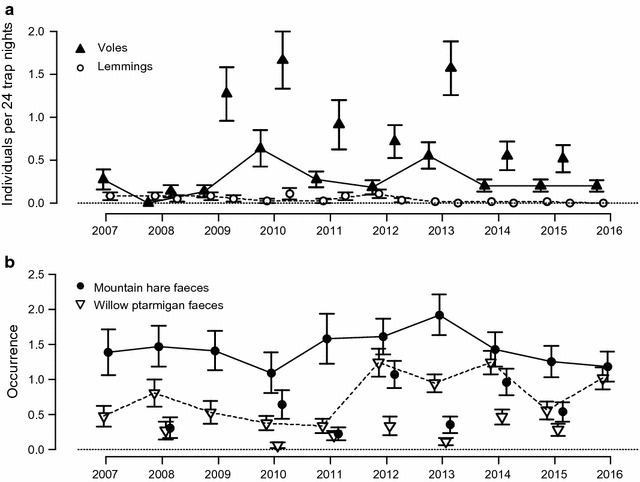
Dynamics of main herbivores from 2007 to 2016 at Erkuta, southern Yamal. **a** Number of voles and lemmings caught on trapping quadrats per 24 trap nights. **b** Occurrence of mountain hare and willow ptarmigan faeces estimated as the number of small plots surrounding a quadrat where faeces were recorded (max eight small plots). For each trapping/faeces counting session (second part of June and August each year except in 2007, where one observation was carried out in July, in 2009 for faeces counts and in 2016, when only the June trapping and counts were carried out), mean results for all habitats are shown with standard error. Lines join the estimates from June

The two dominant small rodent species, the narrow-sculled vole and Middendorff’s vole, exhibited low amplitude multiannual density fluctuations and reached relative abundance peaks in 2010 and 2013 with 1.67 and 1.38 animals per 24 trap nights in August (Fig. 2a). Abundance always increased over the summer. The three other species occurred at lower densities. Collared lemmings were present in most years, whereas siberian lemmings were nearly absent during the study period. Red-backed voles were rare and possibly increasing [37]. In total, the abundance index fluctuated with a factor 8 in spring and 9 in fall.

The average occurrence of ptarmigan faeces decreased slightly in the beginning of the period, and was highest in the years 2012–2014 (Fig. 2b). Hare faeces occurrence was in general higher and rather stable, with a suggested relative maximum in 2013 (Fig. 2b). The overall high abundance of hare and ptarmigan was corroborated by frequent observations of these species in the study area both in winter and in summer [41].

### Den survey

**Fig. 3:**
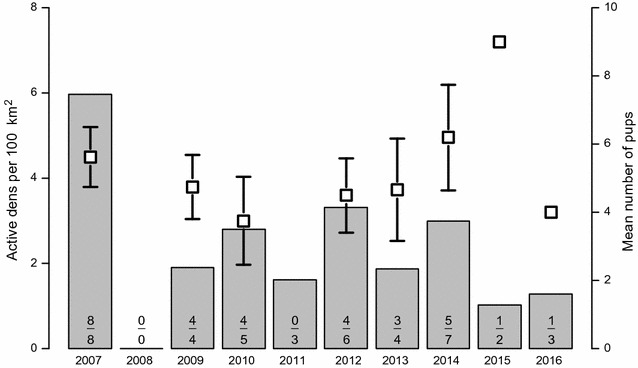
Number of active dens and litter size of Arctic foxes at Erkuta, southern Yamal. Number of active Arctic fox dens (breeding) per 100 km^2^ and mean minimum number of pups with standard errors observed between 2007 and 2016 at Erkuta. The numbers on the bars show the number of active dens per year (lower) and the number of dens where a minimum number of pups could be determined either by proper observation or with automatic cameras (upper)

A total of 59 fox dens were present in the study area (Fig. 1). The smallest den, in which reproduction was observed, had four entrances, thus 47 dens with four or more entrances were considered large enough for breeding. This resulted in an overall density of 17 dens per 100 km^2^ (dens with four entrances or more). Most dens were located on the slopes of hills and often close to lakes. Over the 10 years of the study, 42 breeding events of Arctic foxes were recorded in 23 different dens. In at least four cases, the Arctic foxes moved their pups to a new den in the course of the summer (Fig. 1). The number of active breeding dens was on average 2.6/100 km^2^ and varied between 0 in 2008 and 6 in 2007 (Fig. 3). The proportion of active dens, taking into account only the 25 dens where pups have been observed (23 dens with breeding and two dens to which pups have been moved later in the season), varied between 0 and 0.7. The number of pups observed per den was on average 5.2 (SD = 2.1, maximum 10 pups). Considering each year, the mean number of pups was lowest in 2010 and highest in 2015, when nine pups were observed on the single den where an estimate was obtained (Fig. 3).

**Fig. 4:**
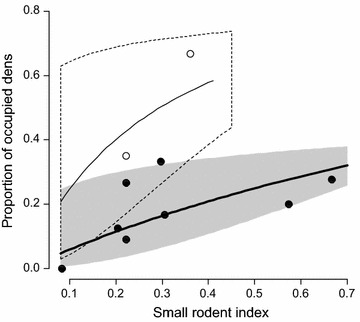
Proportion of active dens in relationship to small rodent abundance and availability of reindeer carcasses. Observed proportion of active Arctic fox dens (breeding) in relation to the small rodent trapping index in June for years with and without high availability of reindeer carcasses for the years 2007 to 2016. Open symbols refer to 2007 and 2014, the years with high availability of reindeer carcasses, whereas filled symbols refer to the other years. Lines show the predicted probability for a den to be active as estimated from a generalized linear mixed model (additive effect of small rodents and reindeer on the logit scale), and polygons show 95% confidence intervals. The thin line and doted polygon refer to years with high availability of reindeer carcasses whereas the thick line and grey polygon refer to the other years

The probability of a den being active in a particular year was best explained by a model with the log of small rodent abundance in June and the availability of reindeer carcasses as additive fixed effects. This model was notably better supported by AICc than models with lemming abundance in June, or lemming abundance in June and the availability of reindeer carcasses as fixed effects (ΔAICc = 12.9 and ΔAICc = 3.1 respectively; Additional file 1: Table S1). Thus, more foxes were breeding after winters with high reindeer mortality (odds ratio = 5.2, 95% confidence interval CI = 2.1–13.7; Table 1; Fig. 4) and in years with higher small rodent abundance in June (odds ratio = 2.8, CI = 1.4–6.6 for an increase of the log of the trapping index by 1).

**Table 1:** Coefficients of a generalized linear mixed effects model for the probability of a den to be active

Coefficient	Estimate	SE	CI
Intercept	−0.39	0.52	
Log (rodents index)	1.03	0.41	0.32; 1.89
Reindeer	1.64	0.46	0.73; 2.62

Random effect of den ID: var = 0.42.  Estimated coefficients of a generalized linear mixed effects model (binomial error distribution) for the probability of an Arctic fox den to be active in a certain year. Fixed effects were the log of the index of small rodent abundance in June and high availability of reindeer carcasses in the previous winter. Den ID was included as random effect on the intercept. Estimates are given on the logit scale with standard error (SE) and 95% bootstrap confidence intervals (CI)

Models including an effect of ptarmigan or hare in addition to small rodents received lower support (Additional file 1: Table S1). For the minimum number of pups per den, the model with an intercept only received most support from AICc, but ΔAICc to the models with the log of small rodent abundance in June or the availability of reindeer carcasses as fixed effects was small (ΔAICc = 0.72 and 0.45 respectively; Additional file 1: Table S2). All three models had substantial Akaike weights (between 0.19 and 0.27), but none of them indicated that the explanatory variables had a strong effect on the number of pups (Additional file 1: Table S3).

### Arctic fox diet

**Fig. 5:**
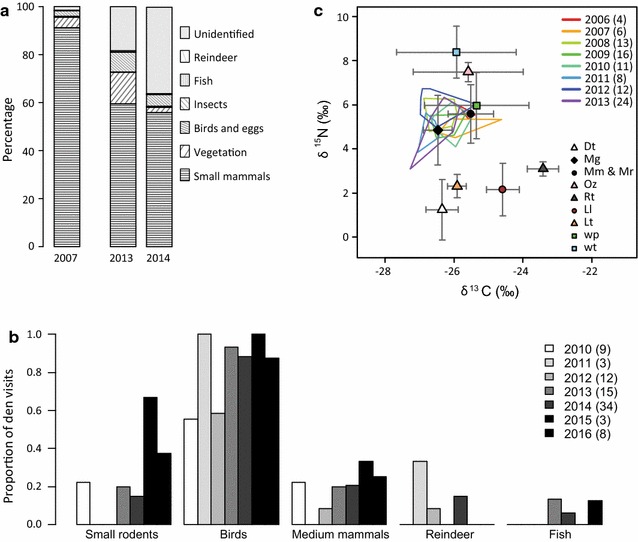
Diet of Arctic foxes at Erkuta, southern Yamal. **a** Percentage of each prey category identified from scat dissection. In 2007 percentage of dry weight was estimated, whereas in 2013 and 2014 percentage of volume was estimated. These estimates can thus not be directly compared. **b** Proportion of den visits in the years 2010–2016 during which remains of different categories of prey were recorded. Number of visits per year are given in parentheses. **c** Polygons surrounding the stable isotope signatures of Arctic fox winter fur are shown in different colours for each year (i.e. the year during which the fur was growing; sample sizes in parentheses). Values have been corrected for isotopic discrimination according to Lecomte et al. [49]. Average signatures of main prey groups are shown with standard deviations: Dt, collared lemming; Mg, narrow-sculled vole; Mm and Mr, Middendorff’s vole and red vole; Oz, muskrat; Rt, domestic reindeer; Ll, willow ptarmigan; Lt, mountain hare; wp, waders and passerines; wt, waterfowl

Rodent remains represented the most important component in Arctic fox scats. This was the case when considering proportions of dry weight (2007), proportions of volume (2013–2014; Fig. 5a), and frequencies of occurrence: Rodent remains were found in the bulk samples from all dens in 2007, in 75% of the scats in 2013 and in 78% of the scats in 2014. As these estimates were based mainly on the amount of fur recovered from the scats, they represented all species of rodents together including fur of muskrat in 2007. In 2013 and 2014 they also included fur of hare. Recovered bone fragments indicated, however, that most fur belonged to small rodents (voles and lemmings). Among recovered small rodent teeth, which could be identified to species, the proportion of lemmings was 48% in 2007 (n = 64; total for all dens), but only 30% in 2013 (n = 43) and 10% in 2014 (n = 57). In addition to small rodents, remains of birds and eggs, plants, insects, hare, reindeer and fish were identified (Fig. 5a). Remains of birds occurred in the bulk samples from all dens in 2007, in 34% of the scats in 2013 and in 18% of the scats in 2014. Except for the proportion of different small rodent species, the proportion of diet components determined in 2007 could not be directly compared to the other years because of differences in methods.

Prey remains were recorded at 16 different dens during up to five visits per den in the course of the same summer. Seven records were from dens visited by foxes but without documented reproduction (not more than one record per den per summer). During 12 visits involving 8 different dens and 9 different breeding events, the observer noted that no prey remains were found. Contrary to the results from scat analyses, birds represented the most common prey remains on dens and were observed during most visits (Fig. 5b). Many of the bird remains belonged to waterfowl (observed at ca 50% of den visits between 2010 and 2013; Additional file 1: Figure S1). Passerines and ptarmigan were also present in all years, although at lower frequencies, whereas remains of waders were rarely identified. Egg shells were observed in 3 of 7 years (Additional file 1: Figure S1). We observed remains of small rodents and hare in 5 years out of seven (Fig. 5b; Additional file 1: Figure S2), and recorded muskrat, reindeer and freshwater fish (northern pike *Esox lucius*) occasionally. The correspondence analysis revealed that there were no consistent differences in prey remain assemblages between the years (Additional file 1: Figure S3). The only year, that was somewhat distinct, was 2015, when only little data were available, but remains of ptarmigan and voles were recorded at two out of three den visits.

The stable isotope signatures of Arctic fox winter fur varied little and there was considerable overlap between years (Fig. 5c). Arctic fox values (corrected for isotopic discrimination) were overlapping with the signatures of voles and small birds (waders and passerines). Moreover, they were located between collared lemmings and hare, and waterfowl and muskrat along the δ^15^N axis, implying that a shift in diet from voles to equal proportions of waterfowl and hare could for instance remain undetected. Overall, the pattern was compatible with a heavy reliance on voles, in particular narrow-sculled voles, and/or with a mixed diet for the population in late summer. The LMM analysis revealed a small but significant decrease in δ^15^N with increasing small rodent abundance (−0.28 ‰ for an increase in one individual per 24 trap nights, CI = −0.51; −0.05), but no effect of small rodent abundance on δ^13^C (Additional file 1: Table S4). Such a shift would be compatible with increased consumption of collared lemmings and a lower proportion of waterfowl in the diet in years with high small rodent densities.

## Discussion

Our analyses showed that small rodents, despite the low abundance of lemmings and the low amplitude fluctuations of the total abundance, were a major resource for Arctic foxes in southern Yamal. They were an important determinant of breeding activity in addition to the availability of reindeer carcasses in winter. Moreover, during the breeding season they were an important component of the diet together with birds.

The discrepancy of the diet composition resulting from scat dissection and prey remains is in agreement with what has been shown previously, notably in studies of the diet of raptors [42]. Larger prey are overrepresented in prey remains, whereas raptor pellets show an exaggerated proportion of small mammal prey [50]. For Arctic foxes, it is likely that the importance of small rodents is underestimated from prey remains, as these are usually consumed rapidly and eaten whole. Birds, on the contrary, may be underrepresented in scat dissection data, as small bone fragments are difficult to identify, and feathers are not consumed in large amounts, but often left outside the fox dens.

The proportion of small rodents inferred from the scat analysis (up to 60% based on volume estimates) was lower than what has been reported with a similar approach for lemming fox populations for instance along the Siberian Arctic coast (76–87%; [15] or in northern Sweden (more than 80%; [14]). The scat analysis data did not allow to address a functional response of the foxes to the small rodent population fluctuations. Stable isotopes, however, revealed a slight decrease in δ^15^N with increasing small rodent abundance, which could be compatible with a small increase in the consumption of lemmings, but could also reflect other diet changes (Fig. 3c). The stable isotope data showed that the signatures of Arctic fox fur at Erkuta varied little compared to other regions in the Arctic [10, 51]. A high degree of similarity in isotopic composition among prey limited, however, the inference of diet we could make using stable isotope data at Erkuta [10]. Together with the high proportion of birds and remains of other prey near the dens, this indicates that Arctic foxes at Erkuta feed opportunistically and rely on a diverse prey base during the summer. There was, however, no evidence for the use of marine resources in any year (see also [10]). Moreover, the analysis of prey remains also showed that the diet of this fox population varied little from year to year, a situation that is rather untypical for inland foxes [23].

At Erkuta, Arctic foxes were breeding in nine out of the 10 years of our study. The number of active dens varied more than in Svalbard, where foxes of the coastal ecotype were breeding every year (11-year study; [19], but less than what is typical for lemming fox populations, such as on Bylot Island [52] or in Scandinavia [53]. The density of active dens was lower than observed on Kolguev Island in a non-rodent population, where breeding occurred every year (6-year study) and the main resource during the breeding season was colonially nesting geese [22]. The reproductive dynamics of the Arctic fox population at Erkuta thus represented an intermediate position along the gradient from highly fluctuating lemming fox populations to more stable non-rodent or coastal fox populations. Therefore, our results, describing an inland mixed-diet vole fox population, support the suggestion of Eide et al. [19] to move beyond the distinction between lemming foxes and coastal foxes.

The probability of a den being active increased with the overall abundance of small rodents, but models including only lemmings received low support (Additional file 1: Table S1). Voles were considerably more abundant than lemmings in our study area (Fig. 2a). Lemmings represented <10% of the small rodents trapped in June in 6 out of 10 years, and in the year when only lemmings were caught in June (2008), Arctic foxes did not breed. This clearly demonstrates that voles (*Microtus* spp.) are a driver of reproductive activity in this Arctic fox population, contrary to results from Fennoscandia, where Arctic foxes responded to lemming densities, but not to voles [5, 15]. It is thus likely that voles are to some degree accessible to Arctic foxes also in winter, contrary to what seems to be the case in Fennoscandia [27]. This might be due to lower amounts of snow than in Fennoscandia (total precipitation during the coldest quarter is 55 mm in Erkuta compared to 120–130 mm for instance on Varanger Peninsula in northeast Norway; http://www.worldclim.org/bioclim), and to different wintering habitats of the various vole species. Accessibility of voles to Arctic foxes in winter has previously been suggested by [54], who reported that about 1/3 of small rodent remains were voles, when considering remains identified in stomach contents from foxes shot in southern Yamal in the winters 1939–1941—a period when lemming outbreaks were occurring in the area.

High availability of reindeer carcasses after winters with icy pastures following ROS events (resulting in bad feeding conditions) also had a positive effect on breeding activity. The resource pulses created by dramatic reindeer mortality during winters 2006–2007 and 2013–2014 benefitted all generalist predators [30], including the Arctic fox, as they do in Svalbard [20, 21]. Together with the varied alternative resources available in summer, notably waterfowl, ptarmigan, waders, hare and muskrat, which provide food for the growing pups, they probably enhanced the productivity of the foxes. For the development of the Arctic fox population on a decadal scale, these resource pulses might have replaced to a certain degree the lemming outbreaks missing since 2000 [38]. In this study, we estimated the availability of reindeer carcasses through a coarse index, nevertheless its effect was clear. In the future, however, the identified relationship should be confirmed using quantitative data on the availability of carcasses in the study area. The absence of a clear effect of our hare and ptarmigan indices on den occupancy may be due to the fact that these species were indeed abundant in the study area in all years [41]. It is thus likely that they contributed to the late winter diet of the foxes in all years, and that a strong decrease in their abundance might lead to a decrease of den occupancy.

With an average of 5.2, litter sizes were only slightly above those observed in coastal foxes in Svalbard [19], but lower than those observed in lemming foxes in Scandinavia in years with increasing or peak lemming densities [53]. Interestingly, the identified determinants of den occupancy did not have any clear effect on litter size, as we expected based on knowledge from lemming fox populations [11]. A similar pattern was however observed in Svalbard, where the number of active dens was related to the availability of reindeer carcasses, but average litter size was not [19]. Data from Bylot Island also showed that lemming abundance affected litter size much less than den occupancy [52]. Litter size estimates obtained on the dens are likely to reflect both the number of embryos, which may be related to resource availability in late winter as is the case for den occupancy, but also early survival of the pups, which is likely to be related to resource availability at the beginning of the summer [11]. They are, however, not estimates of breeding success at the end of the summer. In our study, the faeces indices for ptarmigan and hare reflect the situation in late winter, whereas small rodent abundance in June represents the early summer. The availability of reindeer carcasses is likely to be most important in late winter, but the carcasses are actively used at least until June (own observations) and may provide additional resources during lactation. We lack, however, estimates of the yearly variation in the availability of the diverse alternative resources used by the foxes in summer, notably migratory birds. The absence of correlation between determinants of breeding activity and litter size in several studies may indicate that when foxes in these populations breed, they produce a certain relatively constant number of pups, but probably only a few of them survive if resources during the summer are scarce. True estimates of reproductive success are likely to be mainly correlated with resource availability in summer.

## Conclusions

Contrary to what has been observed in Fennoscandia [5], the Arctic fox population at Erkuta in southern Yamal is a lemming fox population that seems at present to be able to cope with a shift of the small rodent community from high amplitude cyclicity with lemming dominated peaks, to a vole community with low amplitude fluctuations and a consistent population increase over the summer [55]. Such changes in small rodent dynamics have been related to warmer and less stable winters [56, 57]. Due to climate change, it is likely that such winters will occur more frequently [3]. Arctic foxes at Erkuta showed a reproductive response to vole abundance, but reacted as well to the availability of reindeer carcasses in winters with high mortality among domestic reindeer induced by heavy ROS events. The observed flexibility in resource use is in agreement with the generally opportunistic trophic position of the species [23] and observations, for instance, from eastern Greenland, where the fading out of lemming cycles has affected Arctic foxes less than it has affected snowy owls or long-tailed skuas [4]. At Erkuta, such an adaptation to changing resource dynamics may have been facilitated by occasional winters with high availability of reindeer carcasses, which might have replaced lemming outbreaks in creating sporadic resource pulses stimulating high breeding activity among females, as well as by the high abundance of medium sized herbivores such as ptarmigan and hare. Ongoing climate change leading to warmer autumns, shorter winters and more frequent ROS events may, however, in the future also affect these alternative resources negatively as has been shown for instance for ptarmigan on Svalbard [21] and for mountain hare in southern Norway [58]. Thus, the present state of the Arctic fox population at Erkuta may be transient and future climate induced ecosystem changes might be more difficult to cope with.

At present red foxes are rare compared to Arctic foxes in this low Arctic area, but the resource pulses created by reindeer carcasses seem to promote their expansion [30], and an increase in their population may become detrimental to Arctic foxes [26]. Access to constant resource subsidies in winter are a determining factor for the establishment of red fox populations in tundra [59, 60]. As long as winters with high reindeer mortality remain the exception, and Nenets herders generally manage to keep most of their animals alive, red fox expansion may not reach critical levels for Arctic foxes. If winters with ROS events inducing extensive icing become more frequent due to climate warming [3], it is likely that the herders will adapt their seasonal migrations to avoid catastrophic mortality of their herds. Moreover, occasional red foxes appearing in the area are actively controlled by Nenets hunters, who consider them as much more attractive hunting targets than Arctic foxes. Only continued ecosystem-based monitoring will reveal the fate of this southern Arctic fox population in a changing tundra ecosystem.

## Additional file

**Additional file 1: Table S1.** Candidate models evaluated for arctic fox den occupancy. **Table S2.** Candidate models evaluated for for the number of arctic fox pups per litter. **Table S3.** Estimated coefficients of two generalized linear mixed effects models for the minimum number of pups per den. **Table S4.** Estimated coefficients of a linear mixed effects model for the yearly variation in a) δ^13^C and b) δ^15^C values of winter fur of arctic foxes. **Figure S1.** Proportion of den visits each year during which remains of different categories of birds were recorded among prey remains. **Figure S2.** Proportion of den visits each year during which remains of different categories of mammalian prey remains were recorded. **Figure S3.** Results of a correspondence analysis of prey remains recorded at each visit on active dens or dens with clear signs of recent presence of foxes.
