# Supplementary material for: Vole abundance and reindeer carcasses determine breeding activity of Arctic foxes in low Arctic Yamal, Russia
Source: BMC Ecol. 2017 Sep 16;17:32. doi: 10.1186/s12898-017-0142-z (PMC5602845; doi:10.1186/s12898-017-0142-z)
Supplement: Supplementary file 1 — Additional file 1: Table S1. Candidate models evaluated for arctic fox den occupancy. Table S2. Candidate models evaluated for for the number of arctic fox pups per litter. Table S3. Estimated coefficients of two generalized linear mixed effects models for the minimum number of pups per den. Table S4. Estimated coefficients of a linear mixed effects model for the yearly variation in a) δ13C and b) δ15C values of winter fur of arctic foxes. Figure S1. Proportion of den visits each year during which remains of different categories of birds were recorded among prey remains. Figure S2. Proportion of den visits each year during which remains of different categories of mammalian prey remains were recorded. Figure S3. Results of a correspondence analysis of prey remains recorded at each visit on active dens or dens with clear signs of recent presence of foxes. [file 12898_2017_142_MOESM1_ESM.pdf]

Additional Material to the manuscript

Vole abundance and reindeer carcasses determine breeding activity of arctic foxes in low arctic Yamal, Russia

by

Dorothee Ehrich, Maite Cerezo, Anna Y. Rodnikova, Natalya A. Sokolova, Eva Fuglei, Victor G. Shtro and Aleksandr Sokolov

Table S1 Candidate generalized linear mixed models evaluated for arctic fox den occupancy at Erkuta (logit link and binomial error distribution). Fixed effects were the lemming abundance index in June (Lemmings), the natural logarithm of the total abundance index of small rodents in June (Rodents), a factor for presence or absence of a large amount of reindeer carcasses during the previous winter (Reindeer), and faeces counts of hare and ptarmigan in June (Hare / Ptarmigan). Den ID was included as random effect in all models. For each model the fixed effects, the number of parameters (K), the log likelihood (loglik), AICc, the difference in AICc to the best model (ΔAICc) and the AICc weight are shown. The best model is highlighted in bold.

Fixed effects	K	loglik	AICc	ΔAICc	AICc weight
Intercept	2	-97.70	199.47	14.29	0.00
Lemmings	3	-96.00	198.13	12.94	0.00
Rodents	3	-95.08	196.30	11.12	0.00
Lemmings + Reindeer	4	-90.05	188.32	3.14	0.17
**Rodents + Reindeer**	**4**	**-88.48**	**185.18**	**0**	**0.83**
Rodents + Hare	4	-95.07	198.36	13.18	0.00
Rodents + Ptarmigan	4	-94.84	197.90	12.72	0.00

Table S2 Candidate generalized linear mixed models evaluated for the number of arctic fox pups per litter at Erkuta (log link and Poisson error distribution). Fixed effects were the lemming abundance index in June (Lemmings), the natural logarithm of the total abundance index of small rodents in June (Rodents), a factor for presence or absence of a large amount of reindeer carcasses during the previous winter (Reindeer), and faeces counts of hare and ptarmigan in June (Hare / Ptarmigan). Den ID was included as random effect in all models. For each model the fixed effects, the number of parameters (K), the log likelihood (loglik), AICc, the difference in AICc to the best model (ΔAICc) and the AICc weight are shown. The best model is highlighted in bold, and other important models are in shown in italics.

Fixed effects	K	loglik	AICc	ΔAICc	AICc weight
**Intercept**	**2**	**-64.39**	**133.22**	**0**	**0.27**
Lemmings	3	-64.34	135.59	2.37	0.08
Rodents	*3*	*-63.51*	*133.94*	*0.72*	*0.19*
Reindeer	*3*	*-63.37*	*133.67*	*0.45*	*0.20*
Hare	3	-64.39	135.70	2.48	0.08
Ptarmigan	3	-64.34	135.60	2.36	0.08
Rodents + Reindeer	4	-62.83	135.26	2.04	0.10

Table S3 Estimated coefficients of two generalized linear mixed effects models (poisson error distribution) for the minimum number of pups per den. Both models received some support from AICc, but less than an intercept only model (Tab. S2). Fixed effects were a) the index of small rodent abundance in June and b) high availability of reindeer carcasses. Den ID was included as random effect on the intercept. Estimates are given with standard error (SE) and 95% bootstrap confidence intervals (CI).

a)

Coefficient	Estimate	SE	CI
Intercept	1.34	0.25	
Rodents	-0.27	0.21	-0.69; 0.13

Random effect locations: var = 0.00

b)

Coefficient	Estimate	SE	CI
Intercept	1.54	0.12	
Reindeer	0.23	0.16	-0.09; 0.54

Random effect locations: var = 0.00

Table S4 Estimated coefficients of a linear mixed effects model for the yearly variation in a) $\delta^{13}C$ and b) $\delta^{15}C$ values of winter fur of arctic foxes from Erkuta with sampling location as random effect on the intercept and the small rodent trapping index in August as fixed effect. Estimates are given with standard error (SE) and 95% confidence intervals (CI).

a) $\delta^{13}C$

Coefficient	Estimate	SE	CI
Intercept	-24.25	0.10	
Rodent index	0.13	0.08	-0.03; 0.28

Random effect locations: var = 0.08

b) $\delta^{15}N$

Coefficient	Estimate	SE	CI
Intercept	8.97	0.15	
Rodent index	-0.28	0.12	-0.51; -0.05

Random effect locations: var = 0.17

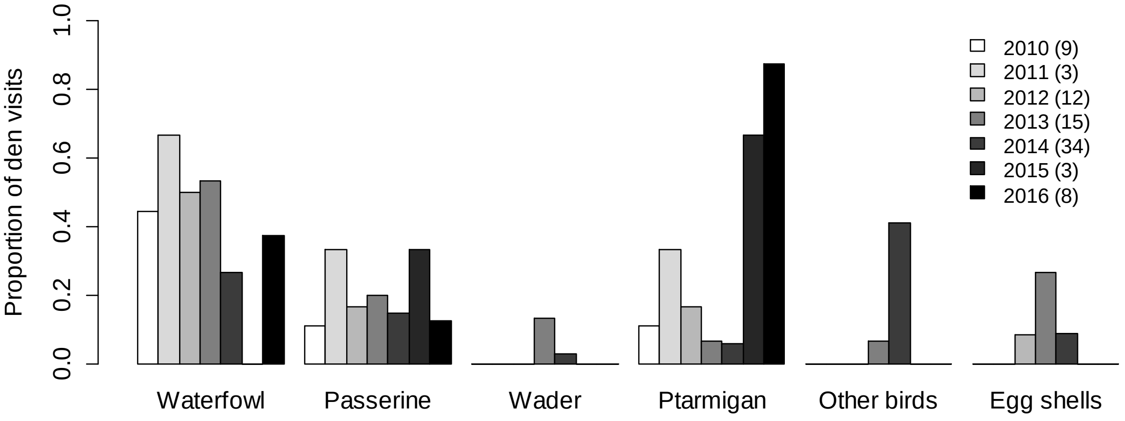

Figure S1 Proportion of den visits each year during which remains of different categories of birds were recorded. Number of visits per year are given in parentheses for each year.

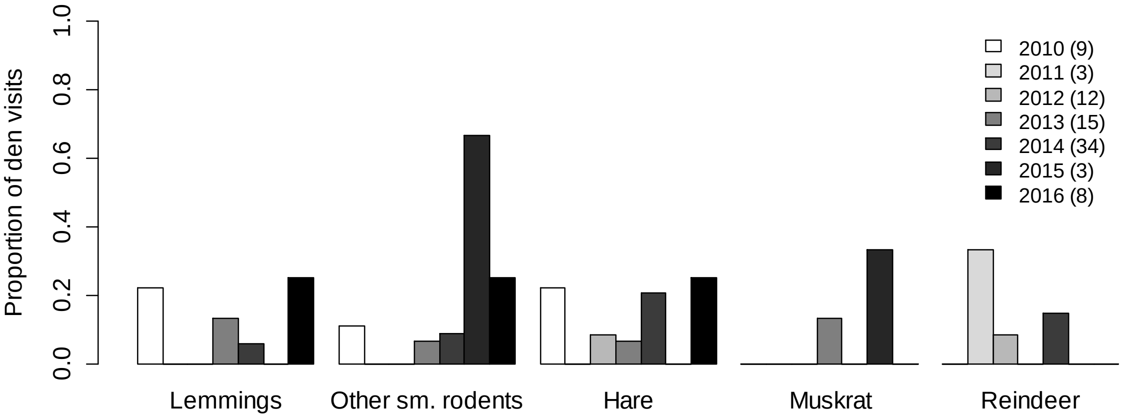

Figure S2 Proportion of den visits each year during which remains of different categories of mammalian prey were recorded. Number of visits per year are given in parentheses for each year.

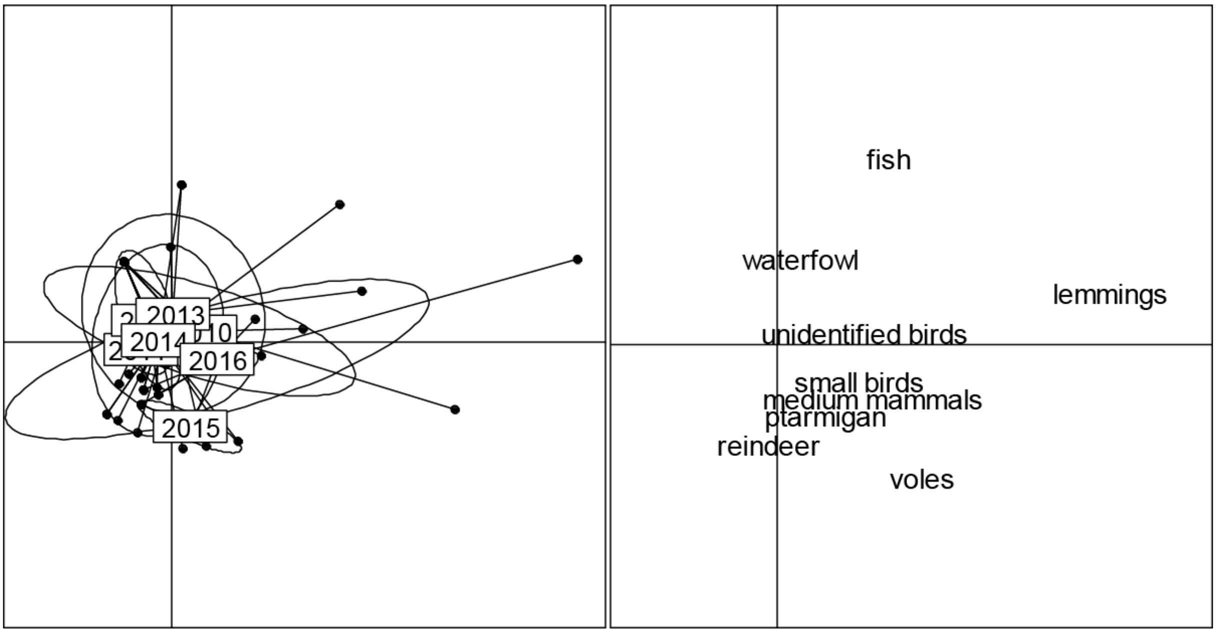

Figure S3 Results of a correspondence analysis of prey remains recorded at each visit on active dens or dens with clear signs of recent presence of foxes. Ellipses on the left plot show approximate 67% confidence intervals around the mean positions for each year.
